# A review on radiation-induced nucleation and growth of colloidal metallic nanoparticles

**DOI:** 10.1186/1556-276X-8-474

**Published:** 2013-11-13

**Authors:** Alam Abedini, Abdul Razak Daud, Muhammad Azmi Abdul Hamid, Norinsan Kamil Othman, Elias Saion

**Affiliations:** 1 School of Applied Physics, Faculty of Science and Technology, Universiti Kebangsaan Malaysia, 43600 UKM Bangi, Selangor, Malaysia; 2 Department of Physics, Faculty of Science, Universiti Putra Malaysia, 43400 UPM Serdang, Selangor, Malaysia

**Keywords:** Radiation-induced method, Metallic nanoparticles, Nucleation, Growth, Redox potential

## Abstract

This review presents an introduction to the synthesis of metallic nanoparticles by radiation-induced method, especially gamma irradiation. This method offers some benefits over the conventional methods because it provides fully reduced and highly pure nanoparticles free from by-products or chemical reducing agents, and is capable of controlling the particle size and structure. The nucleation and growth mechanism of metallic nanoparticles are also discussed. The competition between nucleation and growth process in the formation of nanoparticles can determine the size of nanoparticles which is influenced by certain parameters such as the choice of solvents and stabilizer, the precursor to stabilizer ratio, pH during synthesis, and absorbed dose.

## Review

### Introduction and background

In the past few decades, revolutionary developments of science and engineering have moved at a very fast pace towards synthesis of materials in the nanosize region in order to achieve unique properties that are significantly different from those of the individual atoms and their bulk counterparts [1-3]. When the dimension of a particle decreases below 100 nm, it exhibits many intriguing properties that arise mainly from two physical effects. First, the quantization of electronic states becomes apparent leading to very sensitive size-dependent effects such as optical and magnetic properties [4,5]. Second, the high surface-to-volume ratio alters the thermal, mechanical, and chemical properties of materials [6]. Various nanoparticle synthesis approaches are available, which can be broadly classified into top-down and bottom-up approaches [7]. In the former category, nanoparticles can be obtained by techniques such as milling or lithography which generates small particles from the corresponding bulk materials [8,9]. However, in the latter approach, nanoparticles can be formed atom-by-atom in the gas phase, solid phase, or liquid phase [10]. In the liquid phase, nanoparticles are chemically synthesized in a colloidal solution containing precursors, a reducing agent, a particle capping agent, and a solvent [11,12]. Although colloidal synthesis has the potential to produce large quantity of nanoparticles with good control of size, shape, crystallinity, morphology, composition, and surface chemistry at reasonably low cost.

### Colloidal Metallic Nanoparticles

Colloids are composed of suspensions of one phase, either solid or liquid, in a second liquid phase [13]. They are very attractive because of their huge surface-to-volume ratio and their high specific surface area. This insures contact of a large part of the particle atoms with the surrounding liquid, to form almost as soluble macromolecules, which leads to larger interactions or faster reactions [14]. The colloids, which we are concerned with in this review, are particles of metallic elements with respect to their surrounding phase.

Most of the preparation techniques of the metal colloids are based on reduction of precursor metal ions in solution (aqueous or otherwise) in the presence of a stabilizing agent. The most widely used techniques are thermolysis [15], chemical reduction [16], sonochemical route [17,18], and irradiation methods [19,20]. One of the great advantages of the radiolytic synthesis in comparison with the other available methods lies in the fact that the experiment can be carried out at very mild conditions, such as ambient pressure and room temperature with high reproducibility [21]. Another important advantage of this method is that the main reducing agent in the absence of oxygen is the hydrated electron which has a very negative redox potential. This enables any metal ions to be reduced to zero-valent metal atoms without using chemical reducing agents. Thus, the generation of primary atoms occurs as an independent event and at the origin; the atoms are separated and homogeneously distributed as were the ionic precursors [14,22,23]. In other words, two main factors which lead to formation of uniformly dispersed and highly stable nanoparticles without unwanted by-products of the reductants are homogeneous formation of nuclei and elimination of excessive chemical reducing agents. The choice of the absorbed dose is crucial in order to control the cluster size and crystal structure by precise tuning of nucleation and growth steps especially for multi-metallic clusters [24]. Therefore, the radiation technique has proven to be an environmentally benign and low-cost method for preparation of a large quantity of size and structure controllable metal nanoparticles [24-26].

In this review, a few examples among recent works were selected in which colloidal metal particles were synthesized by radiolytic reduction method and used either as a part of elaborate structures.

## Experimental process

### Radiolytic reduction method

The radiolytic reduction has been proven to be a powerful tool to produce monosized and highly dispersed metallic clusters [25]. The normal ionization radiations which are used for synthesis of nanoparticles are electron beam, X-ray, gamma-ray, and UV light. The metallic nanoparticles can be prepared in an aqueous solution in the presence of a stabilizer without using chemical reducing agents, namely by using of radiolytic method [26-29].

Large number of hydrated electrons $\left(e_{\text{aq}}^{-}\right)$ and H^•^ atoms are produced during radiolysis of aqueous solutions by irradiation (Equation 1). They are strong reducing agents with redox potentials of $E^{0}\left(H_{2}O/e_{\mathrm{aq}}^{-}\right)=-2.87\;V_{\mathrm{NHE}}$ and E^0^ (H^+^/H^•^) = -2.3 V_NHE_, respectively [30]. Therefore, they can reduce metal ions into zero-valent metal particles (Equations 2 and 3).

(1) $$\;H_{2}O\;\overset{\text{Radiation}}{\to }\;e_{\text{aq}}^{-},H_{3}O^{+},H^{•},H_{2},{\mathrm{OH}}^{•},H_{2}O_{2}$$

(2) $$M^{+}+e_{\text{aq}}^{-}\to \;M^{0}$$

(3) $$M^{+}+H^{•}\to \;M^{0}+H^{+}$$

This mechanism avoids the use of additional reducing agents and the following side reactions. Moreover, by varying the dose of the irradiation, the amount of zero-valent nuclei can be controlled.

On the other hand, hydroxyl radicals (OH^•^), induced in radiolysis of water, are also strong reducing agents with E^0^ = (OH^•^/H_2_O) = +2.8 V_NHE_, which could oxidize the ions or the atoms into a higher oxidation state. An OH^•^ radical scavenger, such as primary or secondary alcohols or formate ions, is therefore added into the precursor solutions before irradiation. For example, isopropanol can scavenge OH^•^ and H^•^ radicals and at the same time changes into the secondary radicals, which eventually reduce metal ions (M^+^) into zero-valent atoms (M^0^) as shown in the following reactions [24]:

(4) $${\text{OH}}^{•}+{\text{CH}}_{3}\text{CH}\;\text{OH}\;{\text{CH}}_{3}\to \;H_{2}O+H_{3}{\mathrm{CC}}^{•}\text{OH}\;{\text{CH}}_{3}$$

(5) $$H^{•}+{\text{CH}}_{3}\text{CH}\;\text{OH}\;{\text{CH}}_{3}\to \;H_{2}+H_{3}{\text{CC}}^{•}\text{OH}\;{\text{CH}}_{3}$$

(6) $$M^{+}+H_{3}{\text{CC}}^{•}\text{OH}\;{\mathrm{CH}}_{3}\to \;{\text{CH}}_{3}{\text{COCH}}_{3}+M^{0}+H^{+}$$

**Figure 1:**
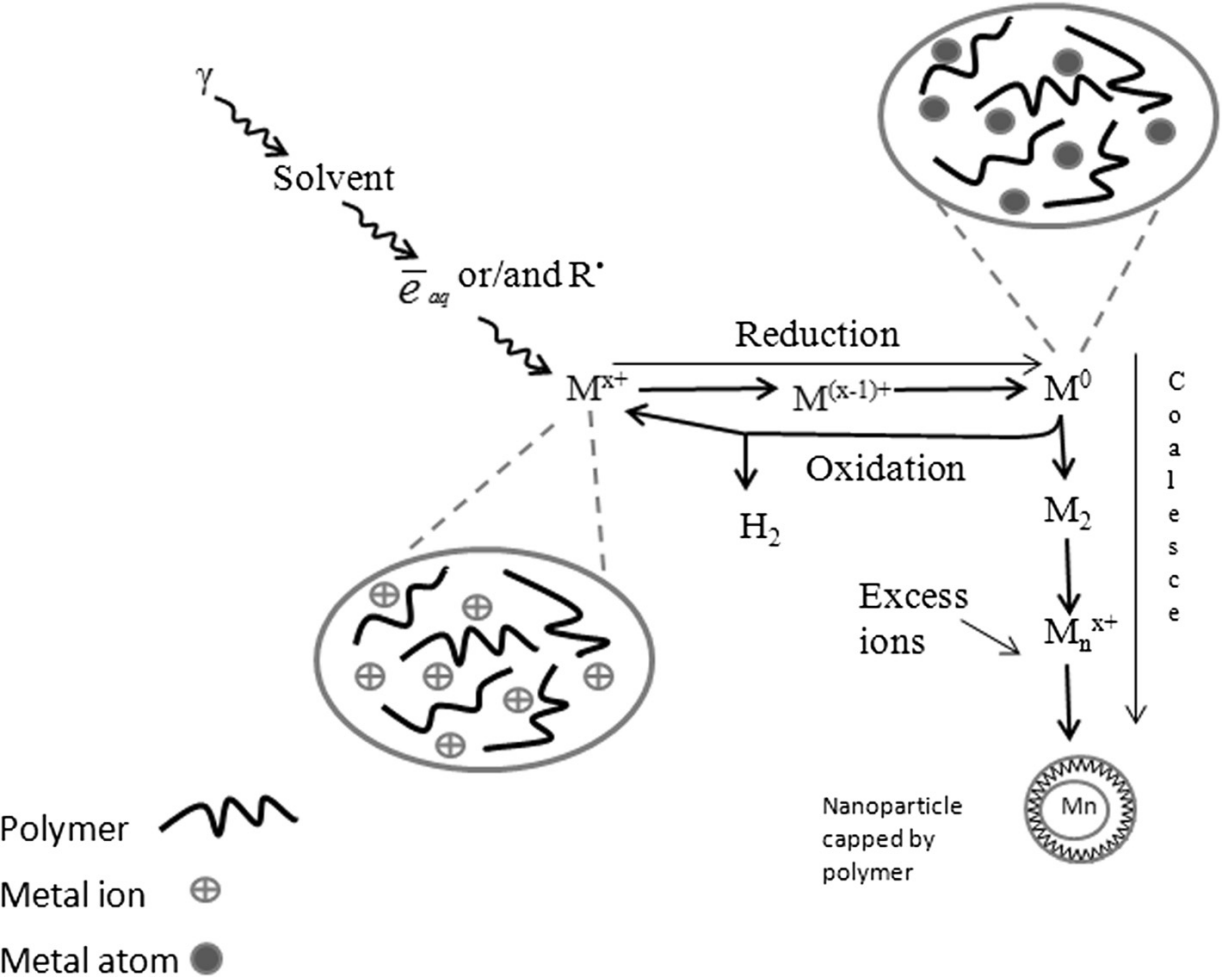
**Scheme of metal ion reduction in solution by ionizing radiation in the presence of stabilizer.** The isolated atoms M^0^ coalesce into clusters. They are stabilized by ligands, polymers, or supports [24].

Multivalent ions are also reduced up to the atoms, by multi-step processes possibly including disproportion of lower valence states. These processes are illustrated by a schematic diagram in Figure 1.

### Nucleation and growth under irradiation

The hydrated electrons arising from the radiolysis of water can easily reduce all metal ions up to the zero-valent atoms (M^0^). Also, the multivalent metal ions could be reduced by multi-step reductions including intermediate valencies. The atoms, which are formed via radiolytic method, are distributed homogeneously throughout the solution. This is as a result of the reducing agents generated by radiation which can deeply penetrate into the sample and randomly reduce the metal ions in the solution. These newly formed atoms act as individual centre of nucleation and further coalescence. The binding energy between two metal atoms or atoms with unreduced ions is stronger than the atom-solvent or atom-ligand bond energy [24]. Therefore, the atoms dimerize when encountering or being associated with the excess metal ions:

(7) $$M^{0}+M^{0}\to \;M_{2}$$

(8) $$\;M^{0}+M^{+}\to \;M_{2}^{+}\;$$

The charged dimer clusters M_2_^+^ may further be reduced to form a centre of cluster nucleation. The competition between the reduction of free metal ions and absorbed ones could be controlled by the rate of reducing agent formation [31].

Reduction of ions which are fixed on the clusters favours to cluster growth rather than formation of new isolated atoms. The bonding between clusters with unreduced ions or two charged clusters is also strong and these association processes are fast:

(9) $$M_{2}^{+}+M_{2}^{+}\to \;M_{4}^{2+}$$

(10) $$M_{m}+M^{+}\to \;M_{m+1}^{+}$$

(11) $$M_{m+x}^{x+}+M_{p+y}^{y+}\to \;M_{n+z}^{z+}$$

where *m*, *n* and *p* represent the nuclearities, and *x*, *y* and *z*, symbolize the number of associated ions. The control of the final size depends on the limitation applied to the coalescence beyond certain nuclearity. For free clusters such as nanocolloids in solution, the coalescence may be limited by a polymeric molecule acting as a cluster stabilizer.

### Stabilization

All nanostructured materials possess a huge surface energy due to the large surface area; thus, they are thermodynamically unstable or metastable. Overcoming the large surface energy to prevent the nanostructures from growing is one of the great challenges in the synthesis of nanomaterials [32]. Nanoparticles, exclusively colloidal particles, in a short distance, are attracted to each other by the van der Waals force. If there is no counteracting force, the particles will aggregate and the colloidal system will be destabilized. The stability is achieved when the repulsion forces balance the attraction forces by electrostatic stabilization and/or steric stabilization.

There are several types of colloidal metal stabilizers which depend on the type of metal, method of preparation, and the application of the resultant metallic nanoparticles. For example, polymers having functional groups such as -NH_2_, -COOH, and -OH have high affinity for metal atoms; however, the use of stabilizers is not desirable for some applications such as catalysis. For example, activities of supported metal nanoparticle catalysts by coordination capture method are higher than those of polyvinyl-pyrrolidone (PVP)-stabilized metal colloidal catalysts [33,34]. Due to functional groups namely C = O and N, and long polymer chains, PVP can associate with the metal nanoparticles [35,36]. The functional groups containing lone pairs of electrons help in the stabilization of metal nanoparticles at their surfaces by covalent interaction, whereas the polymer chain restricts aggregation of metal nanoparticles by steric hindrance. For example, the long chains of PVP stretch out around nickel atom on the surface of the crystal, causing a steric hindrance effect and thus prevent particle growth effectively [37]. Apart from this, PVP is a biocompatible polymer. Hence, nanoparticles synthesized in PVP can be used in biological applications.

There are several reports about using poly(vinyl alcohol) (PVA) as a colloidal stabilizer for the synthesis of metallic nanoparticles by ionizing radiation [38-40]. The PVA chain plays a significant role in avoiding the formation of metal hydroxide clusters by hydrolysis of metal ions, thus preventing them from aggregation. Several active -OH groups in PVA are capable of absorbing metal ions through secondary bonds and steric entrapment [41]. A reaction of metal ions (M^+^) with PVA that leads to their associations can be expressed as:

(12) $$R-\text{OH}+M^{+}\to R-O-M+H^{+}$$

where R-OH represents a PVA monomer.

In the absence of a radical scavenger, the radiation crosslinking of PVA molecules is known to be induced mainly by OH radicals in aqueous medium as shown in Equations 13 and 14 [42]:

(13) $$\text{PVA}\;\left(H\right)+{\mathrm{OH}}^{•}\;\to \;{\mathrm{PVA}}^{•}+H_{2}O\;$$

(14) $$2{\mathrm{PVA}}^{•}\;\to \;\mathrm{PVA}‒\mathrm{PVA}\;\left(\text{Crosslinked}\;\text{polymer}\right)$$

The hydroxyl radicals almost exclusively react with PVA and the reduction of metal ions can take place both by hydrated electrons and the polymeric radicals PVA^•^.

The interactions between the surface of Ag colloids prepared by γ-irradiation and organic molecules containing ethanol and C_12_H_25_NaSO_4_ were discussed by Wang and his group [43]. It was observed that these molecules can restrain the growth of Ag particles and produce a dendrite pattern. The interaction of metallic surfaces with the solvent makes the surfaces become homogeneous; thus, Ag particles lost the anisotropy which played an important role in the formation of dendritic patterns.

Another kind of stabilizer for metallic nanoparticles is inorganic compounds such as metal oxides. They were originally used as catalyst supports. The catalysts are generally transition noble metals (Pt, Re, Rh, etc.) supported on various oxides. For example, Al_2_O_3_ supported Ni nanocluster was synthesized via γ-irradiation by Keghouche and his co-workers [44]. The solution of Ni(HCOO)_2_ · 7H_2_O, Al_2_O_3_, isopropanol, and ammonium hydroxide was γ-irradiated at a total dose of 100 kGy. Since alumina has an amphoteric character, it can play an important role in the fixation of metal ions.

### Bimetallic Nanoparticles

When a mixed solution of two metal ionic precursors M^+^ and M'^+^ is irradiated, three main types of structures can be identified: intermetallic or alloyed structures, core/shell, and heterostructure [45,46]. The reduction process of ionic solution is controlled by the respective redox potential of metallic ions which is the key factor to determine the structure of resultant particles.

### Alloy, core/shell, and heterostructured nanoparticles

Nanoparticles with alloy structure form when initial reduction reactions follow by mix coalescence and association of atoms and clusters with unreacted ions. These alternate associations and then reduction reactions progressively build bimetallic alloyed clusters [24].

The mechanism of alloyed structure formation by radiolysis has been studied in detail, for example for Al^3+^ and Ni^2+^ ionic solution under gamma irradiation by Abedini and her co-workers [47]. Nickel ions can be reduced easier than aluminium ions, and as a result, when the precursor ion solution is irradiated, reduction occurs by successive steps. The unreacted ions are absorbed on the surface of the newly formed clusters to form a charged cluster. These ions then get reduced in situ by hydrated electrons to form alloyed structure.

**Figure 2:**
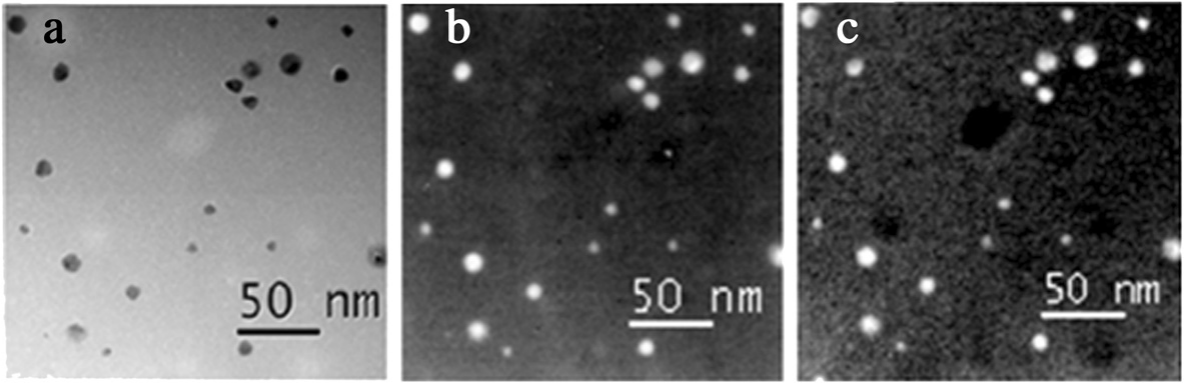
**EFTEM maps of Ag**_**0.9**_**-Ni**_**0.1**_**NPs. (a)** Zero-loss image, **(b)** Ni map, and **(c)** Ag map [48].

Different stoichiometries of Ag-Ni alloy nanoparticles were prepared from an aqueous solution containing AgClO_4_, NiSO_4_, sodium citrate, and methanol, in presence of PVA using the radiolytic method by Nenoff and her co-workers [48]. Figure 2 shows a zero-loss image of Ag-Ni nanoparticles, Ni, and Ag maps by energy-filtered transmission electron microscopy (EFTEM). The shape of bright spots from both Ni and Ag maps is the same which indicates that both Ag and Ni are present in particles with alloyed structure.

**Figure 3:**
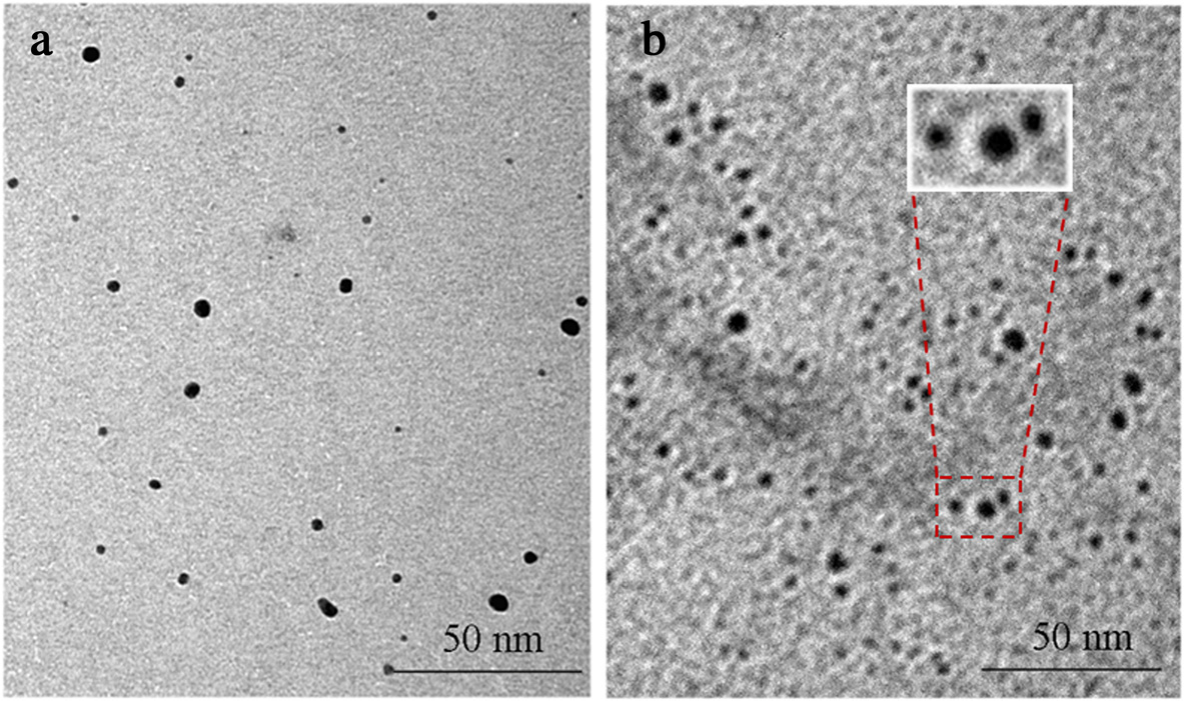
**TEM images of Cu and Cu@CuAlO**_**2**_**-Al**_**2**_**O**_**3**_**nanoparticles. (a)** pure Cu nanoparticles and **(b)** Cu@CuAlO_2_-Al_2_O_3_ nanoparticles in core-shell structure [49].

If the ionic precursors are multivalent and both metals have some probabilities to be reduced by hydrated electrons and radiolytic radicals, the less noble metal ions (M'^+^) will act as electron donors to the more noble metal ions (M^+^). Thus, at the first step, monometallic clusters of noble metal (M_n_) will be formed. Then, when concentration of M^+^ ions decreases, M'^+^ ions are reduced afterwards at the surface of M_n_. The final result is a core-shell cluster where the more noble metal M is coated by the other one M' [24]. For example, the Cu(core)/Al_2_O_3_(shell) nanoparticles were formed when mixed CuCl_2_ and AlCl_3_ solution in the presence of PVP was gamma-irradiated [49]. Copper ions have a higher possibility to be reduced (higher redox potential, E^0^(V) = +0.34) than aluminum ions ( E^0^(V) = -1.66), so the rate of reaction of hydrated electrons in the solution with Cu ions was higher than with Al ions. Thus, when bivalent Cu ions were irradiated, the reduction occurred until Cu zero-valent content increased. Then in a further step, when Cu^2+^ ions were depleted, the reduction of Al^3+^ increased which occurred exclusively at the surface of the Cu particles to form core-shell structure. The core/shell structure of the clusters, as analysed by transmission electron microscopy (TEM; Figure 3), electron diffraction, and XRD, was clearly confirmed [49]. The boundary between the core and shell was not sharp, since the shells are CuAlO_2_ and Al_2_O_3_ instead of pure Al.

**Figure 4:**
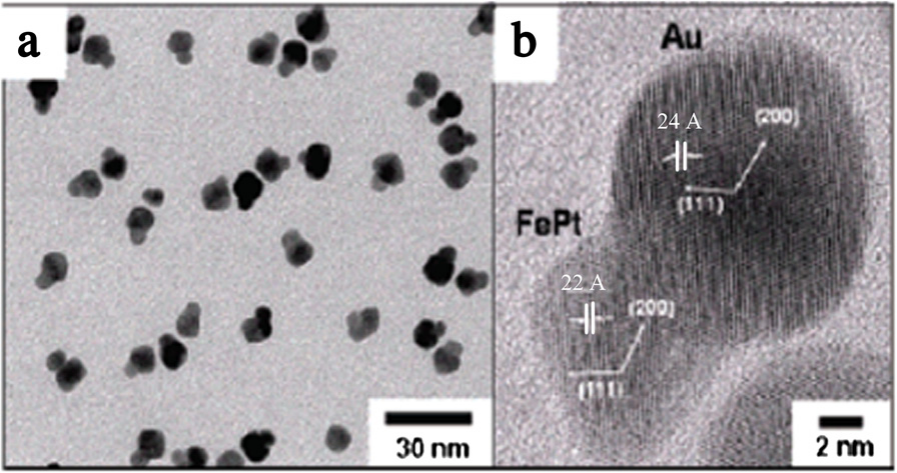
**TEM and HRTEM images of FePt-Au heterostructured nanoparticles. (a)** TEM image, and **(b)** HRTEM image of FePt-Au heterodimer nanoparticles reported by Choi et al. [50].

Under proper conditions, individual nucleation and growth of two kinds of metal atoms can occur separately to form heterostructure. For example, when FePt nanoparticles reacted with AuCl-(PPh_3_) in the presence of 1,2-dichlorobenzene containing 1-hexadecylamine, the successive growth of Au on to the FePt seeds was observed which resulted in the formation of heterodimers of FePt-Au (Figure 4) [50].

### Effects of synthesis parameters

The synthesis of metallic nanoparticles by irradiation is governed by a number of experimental parameters such as the choice of solvent and stabilizer, the precursor to stabilizer ratio, pH value during synthesis, and absorbed dose. All of these parameters determine the final ordering, particle size and distribution, and surface area of resultant nanoparticles. A preliminary study should be done in order to determine the best conditions for an efficient dispersion, and to prepare the further homogeneous fixation of the metal nanoparticles on the support.

### Effect of the solvent type

It has been suggested that the reduction rate under irradiation can be modified by using the appropriate solvent. The reducing agents are the key parameters that can affect the speed of reduction and therefore the particle size and distribution. The hydrated electrons (E^0^ = -2.9 V_NHE_), produced by water radiolysis, are stronger reducing agents than 2-propyl radicals. The existence of different reducing agents in the media varies the speed of reduction that makes a broad size distribution.

Misra and his co-workers [36] have synthesized the Au nanoparticles with narrow size distribution by gamma radiolysis method. They used acetone and 2-propyl alcohol in aqueous media as solvent. Acetone is known to scavenge aqueous electron to give 2-propyl radical (E^0^ = -1.8 V_NHE_) by the following reaction:

(15) $$e_{\mathrm{aq}\;}^{-}+{\mathrm{OH}}^{•}+H^{•}+{\left({\mathrm{CH}}_{3}\right)}_{2}C=O\to {\left({\mathrm{CH}}_{3}\right)}_{2}C^{•}-\mathrm{OH}+{\mathrm{OH}}^{-}$$

**Figure 5:**
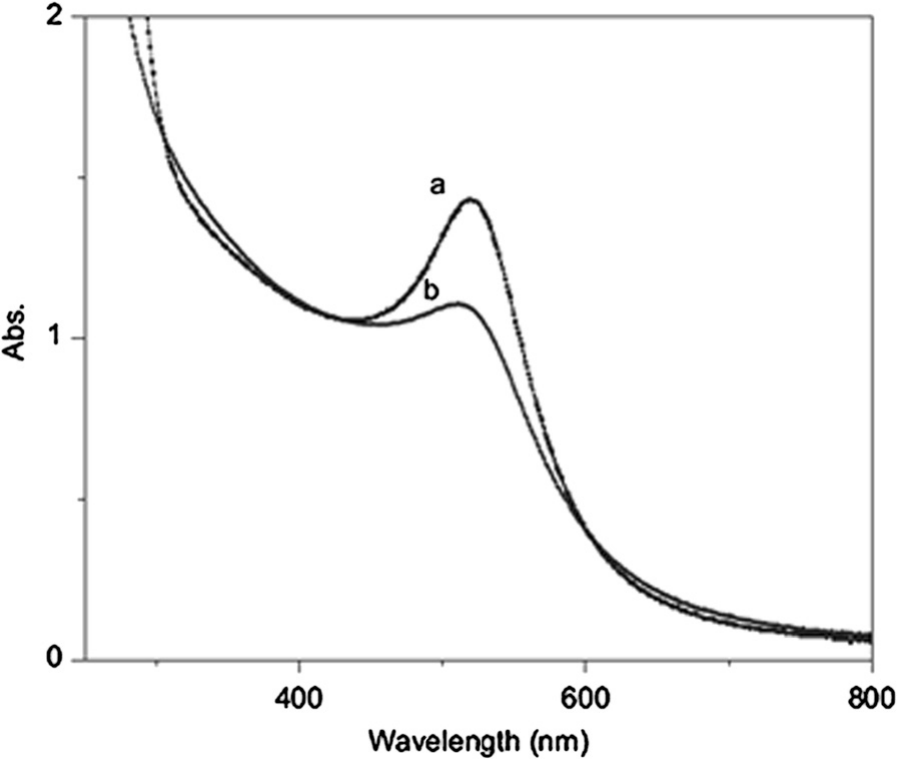
**Absorption spectra of aqueous Au nanoparticle solution.** Absorption spectra obtained (a) with acetone and (b) without acetone for absorbed dose of 1.7 kGy [36].

The only reducing agent in the system is the 2-propyl radical [51]. Reduction by this radical is slower than that by hydrated electron which is suitable for achieving narrower size distribution. It could be clearly observed from Figure 5 that FWHM of absorption peak, which shows size distribution of the particles in a solution, decreases by adding acetone. Also, in the synthesis of Ag nanoparticles by gamma irradiation reported by Mukherjee et al. [52], it has been investigated that as the mole fraction of ethylene glycol in aqueous media increased, the amount of reduced particle increased. The results show the participation of organic radicals in the reduction of silver ions adsorbed over the surface of silver particles.

### Effect of pH of the medium

The optimized pH corresponds to three issues namely, a compromise between the valence state and the charge of ionic precursor in relation with the electrostatic surface charge of the support, preventing reoxidation and minimizing the corrosion of the metallic nanoparticles, and preventing the preparation of unpleasant precipitation. For example, LIU et al. [53] have founded that Cu^2+^ ions in aqueous solution could be oxidized easily when the solution pH was lower than 9.

**Figure 6:**
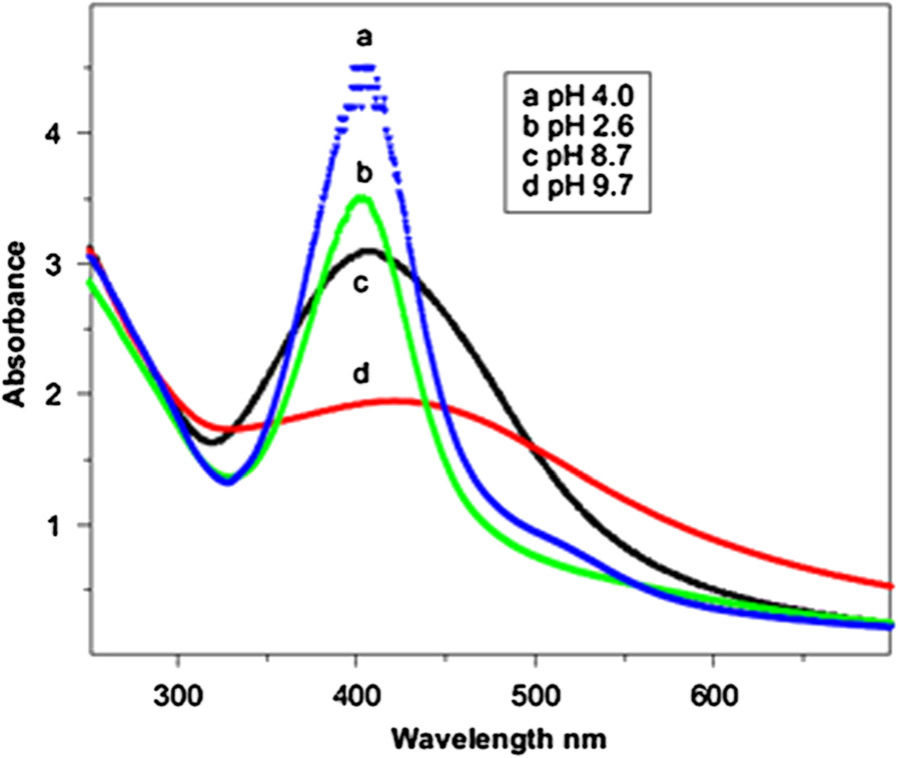
**Radiation-induced formation of Ag nanoparticles on SiO**_**2 **_**at various pH’s: (a) 4.0, (b) 2.6, (c) 8.7 and (d) 9.7; Radiation dose = 0.6 kGy **[54]**.**

Silver nano-clusters on SiO_2_ support have been synthesized in aqueous solution using gamma radiation by Ramnani and co-workers [54]. They observed that, the surface plasmon resonance band, recorded after irradiation, shifts to the red side of the visible spectrum with enhanced broadness when pH was increased (Figure 6). In alkaline media, Ag clusters that formed on the surface of silica were not stable and probably underwent agglomeration. With increasing pH of the irradiated solution, the solubility of SiO_2_ increased and therefore affected stabilization of Ag clusters which resulted in their agglomeration.

### Influence of radiation dose

Nucleation and aggregation processes in the formation of bimetallic nanoparticles could be affected by varying the absorbed dose. The rates of growth could be determined by probabilities of the collisions between several atoms, between one atom and a nucleus, and between two or more nuclei [55]. At low radiation doses, the concentration of unreduced metal ions is higher than the nucleus concentration because of low reduction rate. Thus, the unreduced ions can ionize bimetallic nanoparticles to form large bimetallic ions before they undergo reduction and aggregation processes to form even larger bimetallic nanoparticles. However, at higher doses, most of the metal ions are consumed during the nucleation process; therefore, the nucleus concentration is higher than the concentration of unreduced metal ions. As a result, the bimetallic nanoparticles are smaller in size at higher radiation doses [47].

**Figure 7:**
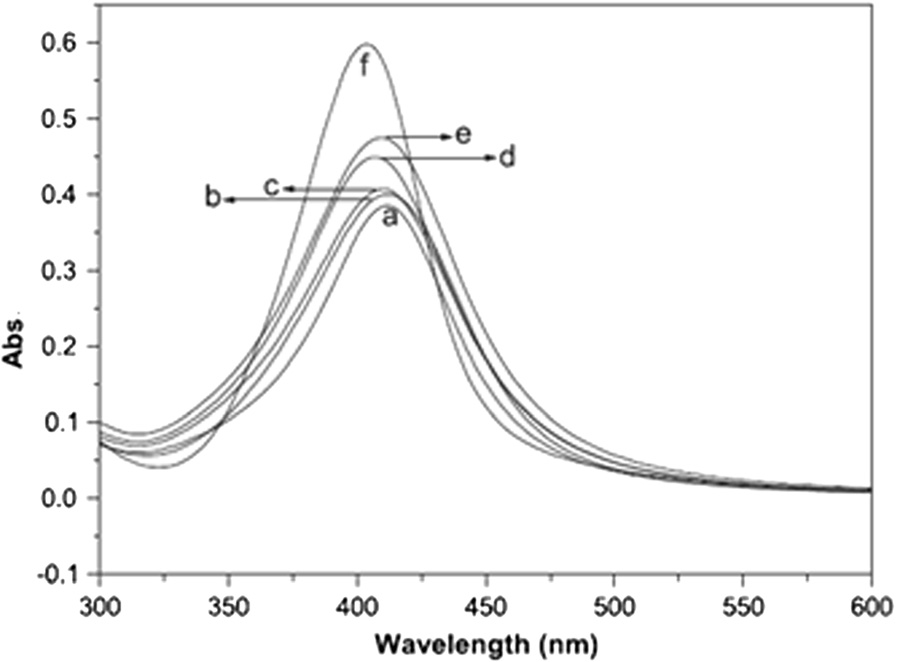
**Optical absorption spectra of silver nanoparticles.** Optical absorption of samples when irradiated at (a) 1.0 kGy, (b) 2.0 kGy, (c) 4.5 kGy, (d) 12.0 kGy, (e) 18.0 kGy and (f) 24 kGy [31].

On the other hand, there is a possibility of inter- and intra-molecular crosslinking within the polymer molecules via radical interaction mechanism as secondary step in gamma-ray reduction. At higher doses, the polymer becomes a more complex matrix due to the occurrence of inter- and intra-molecular hydrogen bonding as well as radical linkage initiated by gamma irradiation between the cyclic structure constituents of the polymer molecules [56]. Therefore, it inhibits the aggregation of colloidal nanoparticles resulting in the formation of smaller nanoparticles. For example, Rau et al. [31], in the synthesis of silver nanoparticles by gamma radiation in the presence of gum acacia, have found that as the irradiation dose increases the corresponding optical absorption intensity increases with concomitant blue shifts. An increase in the intensity of optical absorption spectra indicates the increase of number of silver nanoparticles. In addition, the peak shift may be attributed to the change in particle size (Figure 7).

**Figure 8:**
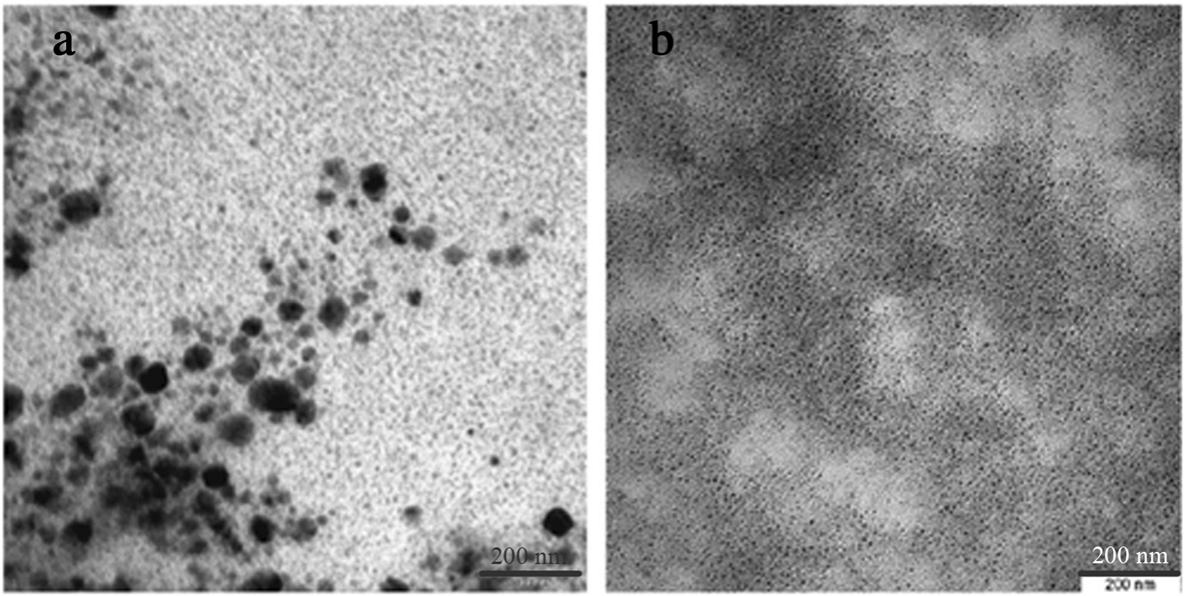
**TEM images of colloidal Al-Ni nanoparticles.** TEM images of Al-Ni nanoparticles at doses of **(a)** 60 kGy and **(b)** 100 kGy [47].

It was reported that the radiation crosslinking of gum acacia molecules can directly affect the growth process of silver nanoparticles [31]. It is important to mention here that we cannot generalize this for all kinds of polymers, for example in contrast with gum acacia, chitosan cannot facilitate the formation of Ag nanoparticles at higher doses and black precipitation was observed at a dose >20 kGy [57]. However, for binary Al-Ni nanoparticles prepared by gamma radiation method the average size of particles decreased from 32.7 nm at 60 kGy dose to 4.4 nm at 100 kGy dose (Figure 8) [47].

A similar trend has been reported for PVP-capped Cu@CuAlO_2_-Al_2_O_3_ nanoparticles synthesized by gamma radiation in aqueous solution at various radiation doses [49]. The average size of Cu@CuAlO_2_-Al_2_O_3_ nanoparticles decreased from 12 nm at 80 kGy to 4.5 nm at 120 kGy. Variation in the particle size could be referred to the difference in the rate of nucleation and growth processes.

### Effect of precursor's concentration

By increasing the initial ion concentration, final size of metal nanoparticles increase [49]. There are three main reasons for the results. Firstly, the rate of ion association that forms larger particles increases by increasing the concentration of metal ions. Secondly, particle aggregation occurs by collision of small particle in solution. The viscosity of the aqueous solution and subsequently the speed of particles movement can be changed by varying the ratio of polymer to ions. Increasing the concentration increases the number of ions and collision probability. Finally, the surface energy and further agglomeration of nanoparticles can be reduced by the adsorption of polymer molecules on the surface of metal nanoparticles [58,59]. Therefore, increasing ion concentration reduces the polymer capping performance on the surface of nanoparticles which leads to the formation of larger particles.

**Figure 9:**
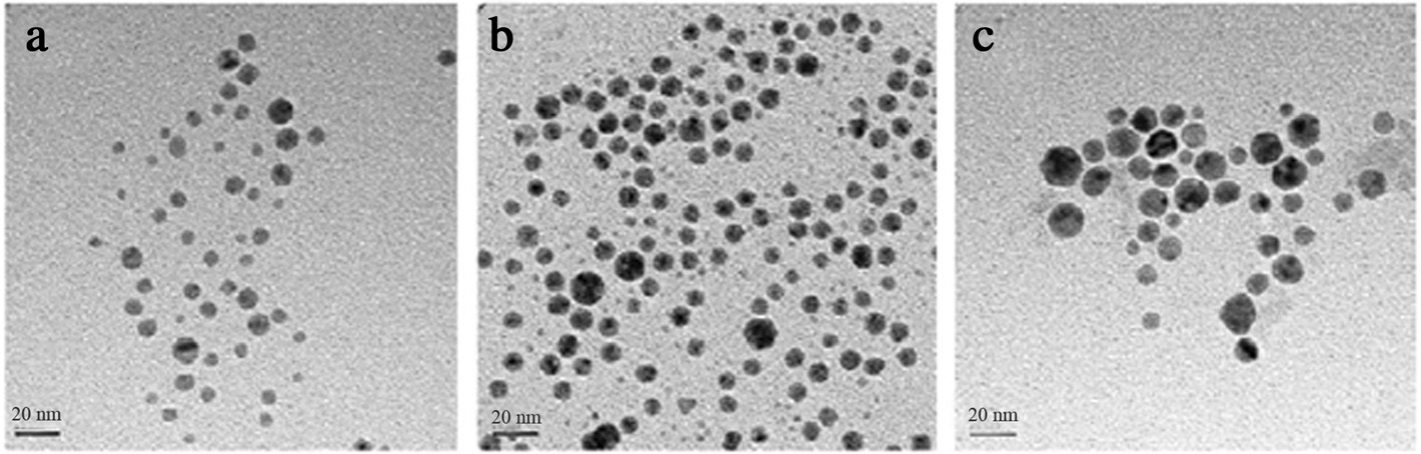
**TEM images of gold nanoparticles.** TEM images of gold nanoparticles prepared by γ-irradiation at various concentration of HAuCl_4_: **(a)** 2 × 10^-4^, **(b)** 1 × 10^-3^, and **(c)** 2 × 10^-3^ M [60].

Li et al. [60] have synthesized silver and gold nanoparticles from aqueous solution of AgNO_3_ and HAuCl_4_ in the presence of 2-propanol and PVP by gamma irradiation method. TEM results showed the average size of Au nanoparticles increased from 7 nm at the lowest ion concentration (2 × 10^-4^ M) to 15 nm at the highest (2 × 10^-3^ M) (Figure 9).

**Figure 10:**
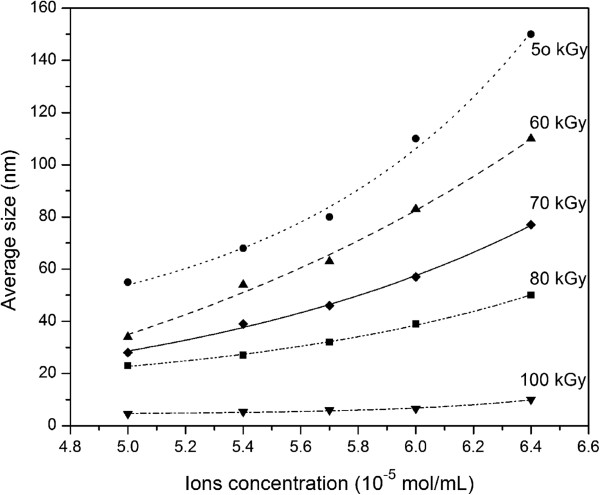
**Exponential trend of changing average size of colloidal Al-Ni nanoparticles versus ion concentration for several doses **[47]**.**

The size of silver and gold nanoparticles increased with the increase in concentration of starting AgNO_3_ and HAuCl_4_ solutions [60]. It indicated that when the number of nuclei remained constant or increased at a slower rate than that of the total ions, the particle size would become larger with the increase of ion concentration. From the data of the UV–vis spectra the irradiation-induced silver colloids from the lowest AgNO_3_ concentration of 2.0 × 10^-4^ M had a light yellow colour with maximum plasmon band at 416 nm. As the concentration of the precursor salt solution increased up to 1.0 × 10^-2^ M, the colour of the silver colloidal solution changed to dark yellow and the absorbance accordingly increased, indicating an increase in the density of resultant Ag nanoparticles formed under irradiation [60]. We could anticipate that the same thing happens to most kinds of bimetallic nanoparticles synthesized by gamma irradiation. Effect of ion concentration on growth process of Al-Ni and Al-Cu bimetallic under gamma irradiation has also been reported [47,49], where the average particle size increased with increasing ion concentration and with decreasing dose (Figure 10).

## Conclusion

In this review, we have surveyed the radiation-induced synthesis and the characterization studies of metallic nanoparticles especially prepared by gamma irradiation. It has been illustrated that the type of solvent, solution pH, precursors' concentration, and the absorbed dose do influence the composition, crystalline structure, particle size, size distribution, and optical properties of the final products. These effects are due to the variation in the nucleation, growth, and aggregation processes in the formation of colloidal metallic nanoparticles. This information could be useful in describing underlying principles in controlling the size of metal nanoparticles by analyzing different combinations of physical factors in monometallic and bimetallic nanoparticle formation.

## Competing interests

The authors declare that they have no competing interests.

## Authors' contributions

AA collected and reviewed the data and drafted the manuscript. ARD and MAAH modified the draft in first version and after revision. NKO participated in the discussion. ES participated in analysis and interpretation of data. All authors read and approved the final manuscript.
